# Phenotyping of acute heart failure with preserved ejection fraction: real‐world outcomes in a cohort of older patients

**DOI:** 10.1111/imj.70324

**Published:** 2026-01-10

**Authors:** Maria Livia Burzo, Giuseppe De Matteis, Amato Serra, Davide Antonio Della Polla, Mariella Fuorlo, Maria Anna Nicolazzi, Benedetta Simeoni, Antonio Gasbarrini, Francesco Franceschi, Giovanni Gambassi, Marcello Covino

**Affiliations:** ^1^ Division of Internal Medicine Ospedale Santo Spirito in Sassia Rome Italy; ^2^ Department of Internal Medicine Fondazione Policlinico Universitario A.Gemelli IRCCS Rome Italy; ^3^ Department of Emergency Medicine Fondazione Policlinico Universitario A.Gemelli IRCCS Rome Italy; ^4^ Università Cattolica del Sacro Cuore Rome Italy; ^5^ Department of Medicine and Translational Surgery Università Cattolica del Sacro Cuore Rome Italy

**Keywords:** heart failure with preserved ejection fraction, acute heart failure, phenotyping, older patients

## Abstract

**Background:**

Previous studies have identified distinct subgroups (phenogroups) of patients with heart failure with preserved ejection fraction (HFpEF).

**Aims:**

This study aims to identify distinct phenotypes in older patients with HFpEF hospitalised for acute heart failure (AHF) and investigate the relationship between subgroups and outcomes.

**Methods:**

Retrospective, single‐center study, including patients ≥65 years hospitalised for AHF over a 4‐year period. We used electronic medical records to collect clinical data, including hospital outcomes. Latent class analysis (LCA) was performed to identify clusters of clinical phenogroups. The primary outcome was all‐cause in‐hospital mortality.

**Results:**

Overall, 770 patients were included. Based on LCA, three phenogroups were identified. Phenogroup 1 (*n* = 323) had both the lowest burden of comorbidities and N‐terminal pro‐brain natriuretic peptides (NT proBNP) values. Phenogroup 2 (*n* = 224) had the oldest patients (median age 82 years), the highest prevalence of women (62%) and atrial fibrillation and the worst right ventricular function. Phenogroup 3 (*n* = 223) consisted mainly of men (57%) and had a higher prevalence of diabetes, obesity and established cardiovascular disease and the worst renal function. Phenogroups 2 and 3 showed a significantly higher risk of primary outcome than phenogroup 1. In addition, survival analysis showed that phenogroup 2 had the worst prognosis, with more than double the risk of in‐hospital death.

**Conclusions:**

In this real‐world cohort of older patients with HFpEF hospitalised for AHF, we identified three subgroups with significantly different features and prognoses. Phenomapping may be an effective tool to identify individuals most likely to experience adverse outcomes, providing a basis for phenotype‐specific treatment strategies.

## Introduction

Heart failure (HF) with preserved ejection fraction (HFpEF) is an increasingly common condition, especially among older patients, and acute HF (AHF) has become the leading cause of hospitalisation among these individuals.^1^ HFpEF is a complex syndrome, caused by several pathophysiological mechanisms involving the interaction between cardiac structural abnormalities and both cardiovascular (CV) and non‐CV comorbidities, and presents with different clinical manifestations.

A growing body of evidence indicates that the HFpEF spectrum is not only composed of a heterogeneous group of patients merely classified by an ejection fraction (EF) ≥50% but that probably includes multiple biological phenotypes with significant functional and outcomes heterogeneity.^2^, ^3^, ^4^, ^5^, ^6^, ^7^, ^8^ To date, phenomapping studies have identified several clusters/phenogroups with shared features.^9^ In this context, “phenomapping” has emerged as a tool that can enable a personalised treatment approach that leads to improved patient outcomes.^10^, ^11^, ^12^

However, while several recent studies have applied this methodology to patient subgroups with chronic HFpEF^12^, ^13^, ^14^ and have included both young and older patients inherently different in terms of prognosis,^10^, ^11^, ^13^, ^15^ it is still unclear whether the same phenomapping could be applied in a population of older patients with AHF who have HFpEF.

Thus, the present study aims to identify possible distinct phenotypes in a real‐world cohort of older patients with HFpEF hospitalised for AHF and to investigate the association between subgroups and clinical outcomes.

## Methods

### Study population

This is a retrospective single‐center cohort study conducted in a large academic medical center, Fondazione Policlinico Universitario A. Gemelli IRCCS (Rome, Italy). We considered all patients aged ≥65 years consecutively admitted to the emergency department (ED) due to AHF and hospitalised in internal medicine and geriatric wards, over a 4‐year period between 1 January 2016 and 31 December 2019.

The criteria for identifying cases included an admission diagnosis of AHF, either *de novo* or acutely worsening HF, as adjudicated by the emergency physician and based on a set of standardised parameters including clinical symptoms, physical examination, laboratory parameters, biomarkers and radiological findings.

In addition, all cases required AHF coded as the primary diagnosis in the discharge record. Diagnoses at hospital discharge were based on International Classification of Diseases, 10th revision (ICD‐10) codes.

Among these patients, we evaluated only those who underwent a two‐dimensional echocardiogram during the hospital stay and who satisfied the definition of HFpEF according to the European Society of Cardiology guideline.^1^

Patients presenting to the ED with AHF due to acute coronary syndromes and requiring catheter‐based interventions, those with advanced atrioventricular blocks or cardiac tamponade, those with pacemakers or implantable cardioverter‐defibrillators and those admitted to an intensive care unit were excluded from the study.

### Study variables

Data were obtained from electronic medical records. For each patient included in the analysis, we collected demographics and clinical characteristics, data on comorbidities, symptoms at ED presentation and information related to hospital stay, including diagnostic tests, treatment and in‐hospital outcome.

Data included age and gender, clinical characteristics and symptoms presented at ED admission, including heart rate, systolic and diastolic blood pressure, peripheral oxygen saturation, body mass index (BMI), dyspnoea, chest pain, fatigue, peripheral edema, oliguria and fever. The New York Heart Association (NYHA) classification was used to categorise all patients according to HF symptoms severity.

Laboratory test results included in the analysis were N‐terminal pro‐brain natriuretic peptide (NT‐proBNP), haemoglobin (Hb) and creatinine. Included in the final analysis were also echocardiographic findings such as left ventricular EF (LVEF), left ventricular end‐systolic diameter (LVSd), left ventricular end‐diastolic diameter (LVDd), tricuspid annular plane systolic excursion (TAPSE), pulmonary artery systolic pressure (PASP), E/a ratio, E/e′ ratio and left atrial volume index (LAVI).

Comorbidities included hypertension, coronary artery disease (CAD), atrial fibrillation (AF), chronic obstructive pulmonary disease (COPD), diabetes, peripheral artery disease (PAD), cerebrovascular disease (CVD), liver disease, chronic kidney disease (CKD) and dementia. The number of comorbidities and their clinical severity were assessed by the Charlson Comorbidity Index.^16^

### Assignment of clinical phenogroups

Latent class analysis (LCA) was performed to determine clusters of clinical phenogroups.

LCA is a clustering statistical technique that uses finite mixture modelling to classify individuals into mutually exclusive and exhaustive subgroups, maximising within‐group similarities and between‐group differences on the basis of multiple observed characteristics in a population.^17^ Participants were characterised based on age, sex, comorbidities, HF symptoms (according to NYHA class) and physiological parameters at ED admission, BMI, LVEF and laboratory values including NT‐proBNP, Hb and creatinine. These clinical covariates were selected *a priori* based on known associations with adverse outcomes in HFpEF.^18^

The latent class models were compared across successive numbers of subgroups (i.e. phenogroups) to determine the best‐fitting model using the Akaike information criterion (AIC).^17^, ^19^ To have an appropriate balance between goodness‐of‐fit and complexity, the model with the lowest AIC value was chosen to select the final number of clusters.

### Outcome measures

The primary endpoint of the study was all‐cause in‐hospital mortality. We classified as CV deaths those occurring due to terminal HF and cardiogenic shock, acute myocardial infarction, arrhythmias, acute pulmonary embolism, cardiac tamponade and acute cerebrovascular disease. Instead, non‐CV‐related events were deaths occurring due to severe sepsis or septic shock, renal failure, respiratory failure due to primary pulmonary diseases, malignancies and major bleeding with hemorrhagic shock.

The secondary endpoint was the length of hospital stay (LOS) calculated as the time from ED admission to hospital discharge or death.

### Statistical analysis

Categorical variables are presented as numbers and percentages. Continuous normally distributed variables are presented as mean ± standard deviation, non‐normally distributed data are presented as median (interquartile range) and binary or ordinal variables are presented as absolute frequency (%). Parametric variables were compared by the Mann–Whitney *U* test (in case of two groups comparison) and Kruskal–Wallis test (in case of multiple groups comparisons). Categorical variables were compared by chi‐square test (with Fisher test if appropriate).

Significant variables at univariate analysis were entered into a multivariate Cox regression model to identify independent predictors for the evaluated endpoints. To avoid overfitting and overestimation of the parameters, the variables with high collinearity were excluded from the multivariate models. If possible, categorical variables were preferred to continuous. The single items composing cumulative variables (i.e. Charlson Comorbidity Index) were excluded from the model to avoid redundancy. The results of the Cox regression analysis are reported as hazard ratios (HRs) with 95% confidence intervals (95% CIs). Survivor analysis was performed according to the Kaplan–Meier approach.

All data were analysed by SPSS version 26 (IBM). A two‐sided *P* value of ≤0.05 was considered significant in all the analyses.

### Statements of ethics

The investigation conforms to the principles outlined in the Declaration of Helsinki and was approved by the local ethical committee (IRB #0051814/19).

## Results

### Classification of HFpEF in clinical phenogroups

**Figure 1:**
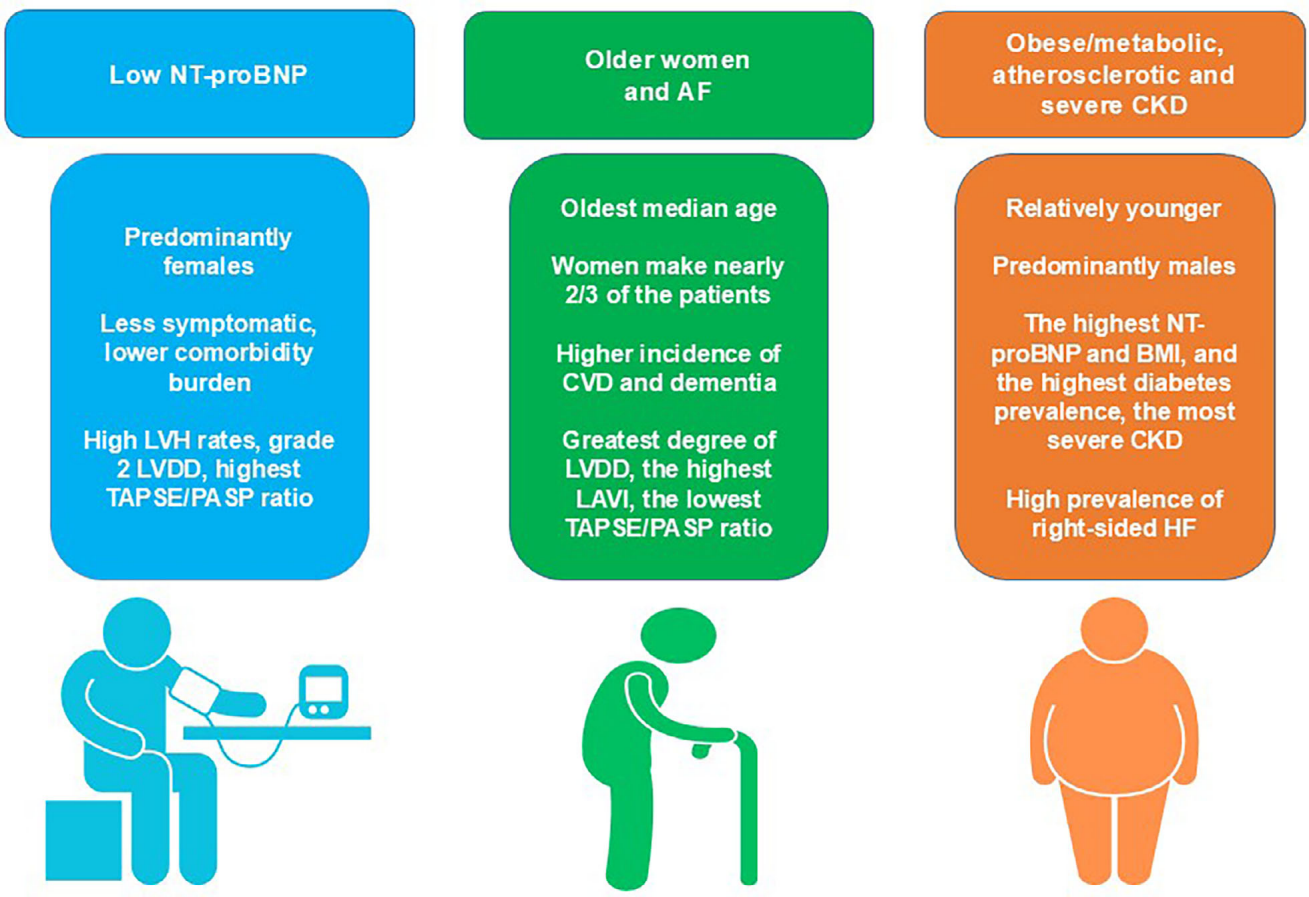
Clinical phenogroups among older patients with HFpEF hospitalised for AHF. AF, atrial fibrillation; AHF, acute heart failure; CKD, chronic kidney disease; CVD, cerebrovascular disease; HF, heart failure; HFpEF, heart failure with preserved ejection fraction; LAVI, left atrial volume index; LVDD, left ventricular diastolic dysfunction; LVH, left ventricular hypertrophy; NT‐proBNP, N‐terminal pro‐brain natriuretic peptide; PASP, pulmonary artery systolic pressure; TAPSE, tricuspid annular plane systolic excursion.

The LCA identified three phenogroups (Fig. 1).

A comparison of baseline characteristics among the three clusters is shown in Table 1.

**Table 1:** Baseline characteristics across clinical phenogroups identified in cluster analysis

	Overall study population (*N* = 770)	Phenogroup 1 (*n* = 323)	Phenogroup 2 (*n* = 224)	Phenogroup 3 (*n* = 223)	*P* value
Demographics					
Age (years)	81 (75–87)	81 (75–87)	82 (77–88)	79 (74–86)	0.01
Male sex	46.8	45.2	38.4	57.4	<0.001
Clinical characteristics on admission					
Heart rate	84 (70–97)	84 (70–96)	87 (72–98)	83 (67–97)	0.72
SBP	132 (112–150)	133 (113–150)	129 (110–145)	135 (120–154)	0.03
DBP	73 (61–83)	73 (63–84)	75 (62–86)	72 (60–80)	0.21
SpO2	93 (91–97)	93 (92–98)	93 (91–97)	93 (91–97)	0.25
BMI (kg/m^2^)	28.5 (23–29)	26.3 (23–29)	27 (23–30)	32 (24–32)	<0.001
NYHA class					
II	19.1	21.1	21.4	13.9	
III	66.2	65.6	64.3	69.1	0.19
IV	14.7	13.3	14.3	17	
Dyspnoea	52.5	44.9	60.3	55.6	0.001
Chest pain	16.2	13	12.5	24.7	<0.001
Fatigue	12.1	10.8	12.5	13.5	0.63
Peripheral oedemas	22.3	20.4	21.9	25.6	0.36
Oliguria	2.5	2.8	2.7	1.8	0.74
Fever	17.1	20.1	17.4	12.6	0.69
Laboratory tests					
NT‐proBNP (pg/mL)	10 800 (2200–13 300)	9200 (1800–12 000	9800 [3300–12 000)	14 000 (2000–18 000)	0.15
Hb (g/dL)	11.2 (9.8–12.5)	11.3 (9.8–12.7)	11.2 (10–12.4)	11 (9.7–12.3)	0.24
Creatinine (mg/dL)	1.6 (0.9–1.9)	1.4 (0.8–1.8)	1.4 (0.9–1.7)	2.1 (1–2.65)	<0.001
Comorbidities					
Charlson Comorbidity Index	6 (5–8)	6 (5–7)	6 (5–8)	7 (6–9)	<0.001
Hypertension	55.1	55.1	53.6	61.4	0.19
CAD	22.1	0	17.4	58.7	<0.001
AF	30.6	0	100	5.4	<0.001
COPD	23.2	20.7	29	21.1	0.05
Diabetes	24.2	0.3	21.4	61.4	<0.001
PAD	14.2%	8	9.8	27.4	<0.001
CVD	5.8	5.3	7.1	5.4	0.61
Liver disease	2.7	2.5	2.2	3.6	0.64
CKD	28.3	24.1	26.3	36.3	0.006
Dementia	5.1	4.6	8	2.7	0.03
Medication at discharge†					
Loop diuretics	85.3	86.2	91.8	78.3	0.002
MRA	51.8	52.3	57.2	46.3	0.133
β‐Blockers	76.4	68.2	81.1	83.4	<0.001
ACEi or ARBs	45.5	49.8	38.4	46.3	0.079
Outcomes					
LOS (days)	11 (5.5–15.3)	11 (5.5–15.4)	11 (5.1–15)	12.7 (6.1–15.3)	0.27
Death	8.3	5	10.7	10.8	**0.01**
CV death	4.6	3.1	6.2	4.9	0.221
Non‐CV death	3.7	1.9	4.4	5.8	**0.049**

*Note*: Values are given as median and interquartile ranges (IQRs) and as percentages for categorical variables.

ACEi, angiotensin‐converting enzyme inhibitor; AF, atrial fibrillation; ARB, angiotensin receptor blocker; BMI, body mass index; CAD, coronary artery disease; CKD, chronic kidney disease; COPD, chronic obstructive pulmonary disease; CV, cardiovascular; CVD, cerebrovascular disease; DBP, diastolic blood pressure; Hb, haemoglobin; LOS, length of hospital stay; MRA, mineralocorticoid receptor antagonist; NT‐proBNP, N‐terminal pro‐brain natriuretic peptide; NYHA, New York Heart Association; PAD, peripheral artery disease; SBP, systolic blood pressure; SpO2, peripheral oxygen saturation.

† Data available for 573 patients of the overall study population (specifically 239 patients for phenogroup 1, 159 patients for phenogroup 2 and 175 patients for phenogroup 3).

Phenogroup 1 individuals (*n* = 232) had a median age of 81 years (75–87 years) and were predominantly women (55%). Patients in this phenogroup presented to ED complaining less frequently of symptoms of dyspnea, fatigue, and peripheral oedemas. Therefore, they were less frequently attributed to NYHA class IV than the other two phenotypes. This phenogroup was also characterised by the highest median LVEF (59% (56–63)), lowest NT proBNP values (median 9200 pg/mL (1800–12 000)), and lowest BMI value among the three clusters. In addition, phenogroup 1 had the lowest comorbidity burden.

Patients in phenogroup 2 (*n* = 224) were mainly women (62 %), had a higher median age (82 years (77–88 years)), and were more likely to have AF. This phenogroup also showed the highest incidence of CVD (7 %) and dementia (8 %).

Phenogroup 3 individuals (*n* = 223) were predominantly men (57 %) with a higher prevalence of diabetes, obesity and established CV disease (e.g. history of CAD and PAD). Patients in phenogroup 3 had the highest NT‐proBNP levels (median 14 000 pg/mL (2000–18 000)) and the worst kidney function (median creatinine value 2.1 mg/dL (1–2.65)). At presentation to ED, patients in phenogroup 3 frequently complained of chest pain. Over 50 % of patients in this group had NYHA class III or IV symptoms.

A summary of the characteristics of the three phenotypic cohorts is illustrated in Figure 1. The three phenogroups were then named: the ‘Low NT‐proBNP group’, the ‘Older women and AF group’ and the ‘Obese/Metabolic, atherosclerotic and severe CKD group’.

### Echocardiographic findings from clinical phenogroups

Echocardiographic findings from clinical phenogroups are presented in Table 2. Phenogroups 1 (74%) and 3 (77%) showed a significantly higher rate of left ventricular hypertrophy (LVH) compared with phenogroup 2 (61%; *P* = 0.006). Furthermore, a statistically significant difference in the TAPSE/PASP ratio was reported among the three phenogroups. The lowest TAPSE/PASP ratio was described in phenogroup 2 (TAPSE/PASP 0.42; *P* < 0.001). In addition, regarding left ventricular diastolic dysfunction (LVDD), more than half of the patients in phenogroups 1 and 3 had grade 2 (57% and 53%, respectively), while patients in phenogroup 2 had more frequently grade 3 (15%; *P* = 0.002).

**Table 2:** Echocardiographic data across clinical phenogroups identified in cluster analysis

	Phenogroup 1	Phenogroup 2	Phenogroup 3		*P*‐value
Echocardiographic findings					
LVEF	59 (56–63)	57 (54–60)	57 (54–61)		<0.001
Left ventricular hypertrophy	74.4	61.4	76.9		0.006
LVDd (mm)	47 (41–52)	47 (41–52)	47 (42–51)		0.819
LVSd (mm)	30 (26–35)	31 (26–35)	31 (26–34)		0.800
TAPSE (mm)	20 (18–23)	18 (16–20)	19 (17–24)		<0.001
PASP (mm Hg)	35 (30–45)	40 (35–50)	40 (30–50)		0.021
TAPSE/PASP	0.55 (0.40–0.66)	0.42 (0.32–0.52)	0.51 (0.37–0.68)		<0.001
E/A	0.77 (0.61–0.97)	0.80 (0.70–1.41)	0.79 (0.60–1.22)		0.184
E/e′	10 (8.7–14)	12.5 (7.75–15.2)	11 (8–15)		0.264
LAVI (mL/m^2^)	55 (43–61)	58 (50–65)	54 (47–61)		0.002
LVDD ultrasound classification					
Normal	14.0	37.0	8.2		
Grade 1	24.3	7.4	30.6		0.002
Grade 2	56.6	40.7	53.1		
Grade 3	5.1	14.8	8.2		

*Note*: Values are given as median and interquartile ranges (IQR) and as % for categorical variables.

LAVI, left atrial volume index; LVDd, left ventricular end‐diastolic diameter; LVDD, left ventricular diastolic dysfunction; LVEF, left ventricular ejection fraction; LVSd, left ventricular end‐systolic diameter; PASP, pulmonary artery systolic pressure; TAPSE, tricuspid annular plane systolic excursion.

### Prognostic relationship between clinical phenotypes and patient outcome

**Figure 2:**
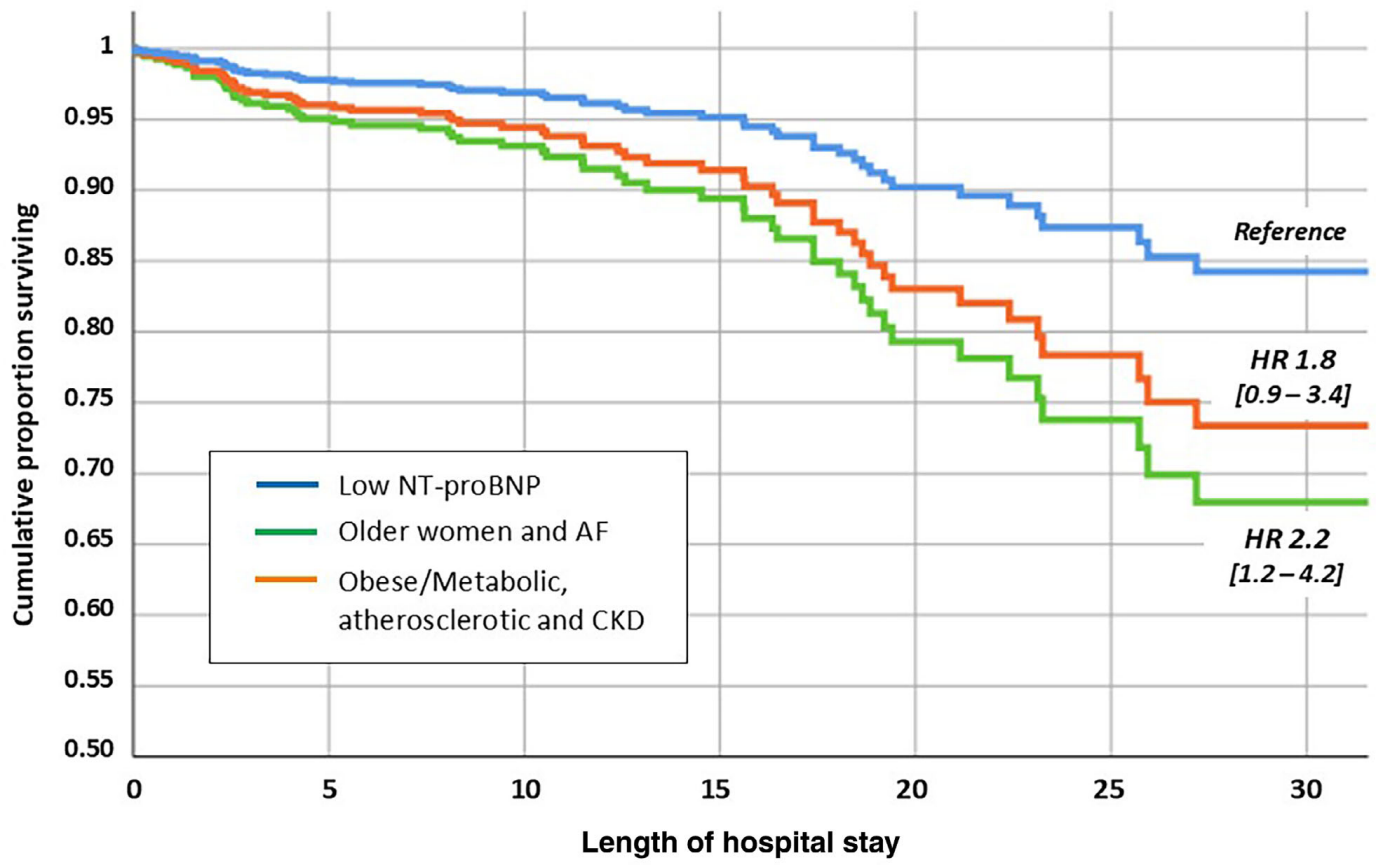
Cox regression analysis for in‐hospital mortality by clinical phenogroups. AF, atrial fibrillation; CKD, chronic kidney disease; HR, hazard ratio; NT‐proBNP, N‐terminal pro‐brain natriuretic peptide.

As shown in Table 1 and Figure 2, phenogroups 2 and 3 exhibited the highest risk of primary endpoint of in‐hospital death compared with phenotype 1 (*P* = 0.01). Among phenotypes with poorer prognosis, as shown in Cox regression analysis, older women with AF revealed the poorest prognosis with a more than 2‐fold increased risk of in‐hospital death (Fig. 2).

**Figure 3:**
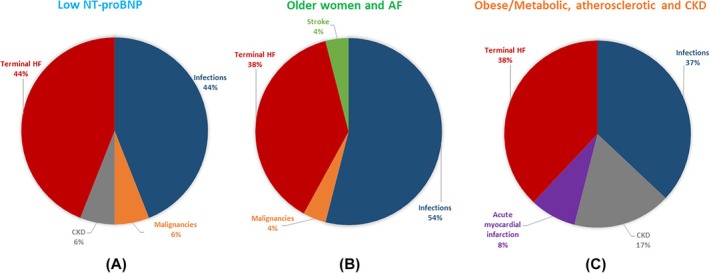
Representation of CV and non‐CV causes of death by clinical phenogroups. Panel A: ‘Low NT‐proBNP’ group; panel B: ‘Older women and AF’ group; panel C: ‘Obese/Metabolic, atherosclerotic and severe CKD’ group. AF, atrial fibrillation; CKD, chronic kidney disease; HF, heart failure; NT‐proBNP, N‐terminal pro‐brain natriuretic peptide.

Differences in mortality from CV and non‐CV causes among the phenogroups are depicted in Figure 3. As shown, infections and terminal HF were the main causes of death among the phenogroups. Furthermore, we found that phenogroup 3 had the highest occurrence of non‐CV death. Expanding on the cause‐specific mortality patterns presented in Figure 3, potential associations between clinical/echocardiographic factors and patterns of CV and non‐CV mortality across phenogroups are summarised in Table S1.

As a secondary endpoint, no differences were observed in LOS among the phenogroups.

## Discussion

In this real‐world, retrospective study of older patients with HFpEF hospitalised for AHF, we identified three distinct HFpEF phenogroups. Based on their unique characteristics, these phenogroups were categorised into Low NT‐proBNP, Older women and AF and ‘Obese/Metabolic, atherosclerotic and severe CKD groups. Given the substantial differences in outcomes among these phenogroups, our findings suggest the potential for stratifying older patients with acute HFpEF into distinct clinical phenotypes, paving the way for phenotype‐specific therapeutic strategies.

### ‘Low NT‐proBNP’ phenogroup

In the low‐NT‐proBNP phenogroup, patients had a median age of 81 years and were predominantly women. Compared with other phenogroups, patients were less symptomatic with the lowest comorbidity burden as well as the lowest NT‐proBNP values. As for echocardiographic features, patients included in this group showed high rates of LVH (74 %), grade 2 LVDD (57 %) and the highest TAPSE/PASP ratio (0.55).

This group has already been described in HFpEF phenomapping studies and it is estimated to account for 15% to 35% of the HFpEF patient population.^14^, ^20^, ^21^, ^22^ Sabbah *et al*.^22^ described a ‘non‐inflammatory’ phenotype characterised by low NT‐proBNP values, lower fibrosis and inflammatory biomarkers and more favourable markers of HF severity. Similarly, Saha *et al*. in a cohort of patients with HFpEF hospitalised for HF described a phenogroup of younger individuals with low NT‐proBNP values, lower burden of comorbidities and better prognosis.^14^ Morevoer, in the phenotyping study of HFpEF derived from the analysis of participants in TOPCAT (Treatment of Preserved Cardiac Function Heart Failure With an Aldosterone Antagonist Trial), Cohen *et al*.^20^ identified a phenogroup with a lower proportion of atherosclerotic risk factors and AF. As reported in the analysis, this group showed a better prognosis for in‐hospital death and the composite of all‐cause death and hospital readmissions.

Notably, patients in the low NT‐proBNP phenogroup of the present study are not completely aligned with those described in the literature, who are often obese and relatively young.^14^, ^22^ However, the median age of the overall population included in our analysis was relatively higher than that reported in previous studies on HFpEF describing this phenotype.^14^, ^20^, ^21^, ^22^

In agreement with these favourable features, in the outcome analysis this group presented the lowest risk of in‐hospital death compared with other phenogroups. Consequently, this phenogroup could be classified as low risk with the best outcome among older patients with acute HFpEF.

### Older women and AF phenogroup

The older women and AF group of the present study had the oldest median age (82 years), and women comprised nearly two‐thirds of the patients. Patients in this phenogroup had a higher incidence of CVD (7%) and dementia (8%). Among these, HF appears to be distinctively more severe, with the greatest degree of LVDD, the highest LAVI, the worst right ventricular function and the highest event rates compared with other phenogroups, mainly due to CV deaths.

Within the comorbidity spectrum of patients with HFpEF, AF represents the most common arrhythmia, occurring in at least one‐third of all patients, being particularly prevalent among older women.^23^, ^24^ In the *post hoc* analysis of data from I‐PRESERVE (Irbesartan in Heart Failure With Preserved Ejection Fraction Study) and CHARM (Candesartan in Heart Failure: Assessment of Reduction in Mortality and Morbidity‐Preserved) study,^12^ Kao *et al*. used LCA to identify six clinical subgroups of patients with HFpEF. One of the subgroups, predominantly composed of older individuals with the highest prevalence of AF, was consistent with our older women and AF phenogroup in terms of both clinical characteristics and prognosis. Similarly, among the three phenogroups identified in the TOPCAT analysis, Cohen *et al*.^20^ described a phenotype characterised by patients of older age with AF, who presented with an elevated risk of in‐hospital death and rehospitalization for AHF. Furthermore, in a study of 365 patients with HFpEF derived from NARA‐HF (Nara Registry and Analysis for Heart Failure),^25^ Kyodo *et al*. used hierarchical modelling to identify three phenotypes among those hospitalised for acute decompensated HF. Among them, the authors described one cluster consisting of women with AF. This group had a poor prognosis at the primary endpoint of in‐hospital death and at the secondary endpoint of death from all causes and readmission for HF within 5 years of follow‐up.

A strong correlation between AF and HFpEF has previously been described, but studies are still ongoing to clarify its prognostic role in HFpEF outcomes. However, the HFpEF phenotype of older women with AF seems to be the most clearly identified phenotype in the most recent HF reports and has consistently been associated with a higher event rate.^20^, ^26^, ^27^ Moreover, the ecochardiographic features of this phenogroup of patients with dilated hearts, lower EF and associated right ventricular dysfunction seem to describe an ‘HFrEF‐like' phenogroup.

### Obese/Metabolic, atherosclerotic and severe CKD phenogroup

The Obese/Metabolic, atherosclerotic and severe CKD phenogroup included predominantly men and relatively younger individuals. Patients in this phenogroup had the highest NT‐proBNP levels and, notably, both the highest diabetes and CKD rates. In addition, these patients had a higher rate of right‐sided HF and a less favourable survival outcome than the low NT‐proBNP phenogroup.

Phenogroups with a higher incidence of atherosclerotic risk factors have also been identified in previous reports. Among the subgroups identified by Kao *et al*.^12^ applying the LCA to the cohort of patients enrolled in the I‐PRESERVE and CHARM‐Preserved trials, one of the two subgroups with the worst event‐free survival in both studies was characterised by a high prevalence of obesity, hyperlipidemia, diabetes, anaemia and renal insufficiency. A subgroup of patients with HFpEF demonstrating more functional impairment, obesity, diabetes, CKD and concentric LVH and the highest risk of the primary endpoint of CV death and HF hospitalisation was recently identified among patients enrolled in the TOPCAT trial.^20^ Moreover, a similar phenogroup of patients with high NT‐proBNP levels and relatively low EF was also described in studies by Kyodo *et al*.,^25^ Hahn *et al*.^28^ and Jones *et al*.^29^

This excess of adverse outcomes may be attributed to the high prevalence of diabetes and CKD in these patients, which may result in chronic systemic inflammation, endothelial dysfunction and cardiac hypertrophy, with consequent worsening HFpEF.^30^ Similarly, despite the high burden of CV comorbidities and the increased occurrence of death from acute myocardial infarction, the Obese/Metabolic, atherosclerotic and severe CKD phenogroup in our cohort showed the highest non‐CV mortality mainly related not only to infections but also to the increased impact of renal failure.

## Limitations

Several limitations of this study should be acknowledged. First, this is a single‐center, retrospective study, enrolling a primarily urban population of patients with acutely decompensated HFpEF and thus generalizability to the whole population of patients with HFpEF might be limited. Second, we did not include some relevant variables, such as biomarkers of myocardial injury and other inflammatory parameters, cardiorespiratory fitness and regional adiposity, limiting the differentiating power between the subgroups. However, we used acute‐phase variables routinely accessible and we believe that this would likely facilitate the clinical application of our model. Furthermore, the present study included one of the oldest populations and with the greatest burden of comorbidities among those described in studies published to date, a population with patients at very high risk. Furthermore, given the retrospective nature of our study, we did not perform an external validation of the identified clusters. A prospective validation analysis would be desirable and should be conducted in future studies to further demonstrate generalizability. Finally, because the number of clusters varies depending on the dataset and clustering method, it is difficult to determine whether the number of clusters currently presented is truly reflective of the clinical variability. It is acknowledged that the selection of variables for phenomapping might be somewhat biased.

## Conclusions

In this real‐world retrospective study of older patients with HFpEF hospitalised for AHF, we identified three distinct phenogroups with different outcomes: Low NT‐proBNP, Older women with AF and ‘Obese/Metabolic, atherosclerotic and severe CKD. Despite some overlap in clinical characteristics, our results suggest that pheno‐mapping could effectively identify individuals at higher risk for adverse events.

These findings highlight the potential to stratify older patients with acute HFpEF into distinct phenotypes, providing a foundation for developing phenotype‐specific therapeutic strategies.

## Supporting information


**Table S1.** CV and Non‐CV Factors and Their Contribution to CV and Non‐CV Death
