# Supplementary material for: Phenotyping of acute heart failure with preserved ejection fraction: real‐world outcomes in a cohort of older patients
Source: Intern Med J. 2026 Jan 10;56(3):373–82. doi: 10.1111/imj.70324 (PMC12978209; doi:10.1111/imj.70324)
Supplement: Supplementary file 1 — Table S1. CV and Non‐CV Factors and Their Contribution to CV and Non‐CV Death [file IMJ-56-373-s001.docx]

**Table S1. CV and Non-CV Factors and their Contribution to CV and Non-CV Death**

| Factor | Phenogroup with highest Prevalence | Prevalence (%) | Associated with | Support from Data |  |
| --- | --- | --- | --- | --- | --- |
|  |  |  |  |  |  |
| CKD | Phenogroup 3 | 36.3% | CV and non-CV death | Elevated creatinine and highest combined mortality (CV 4.9%, non-CV 5.8%) in PG3 |  |
| Diabetes | Phenogroup 3 | 61.4% | CV and non-CV death | Known risk for atherosclerosis and infections; high mortality in PG3 |  |
| COPD | Phenogroup 2 | 29.0% | Non-CV death | Pulmonary disease contributing to infection and respiratory complications |  |
| Dementia | Phenogroup 2 | 8.0% | Non-CV death | Highest prevalence in PG2; PG2 shows elevated non-CV mortality (4.4%) |  |
| CVD | Phenogroup 2 | 7.1% | Non-CV death | PG2 with higher stroke-related mortality |  |
| AF | Phenogroup 2 | 100% | CV death | May contribute to thromboembolic risk and hemodynamic instability in PG2 |  |
| CAD | Phenogroup 3 | 58.7% | CV death | Strong association with atherosclerotic burden and CV mortality in PG3 |  |
| PAD | Phenogroup 3 | 27.4% | CV and non-CV death | Marker of systemic atherosclerosis and increased frailty |  |
|  | |  |  |  |  |
| Low TAPSE/PASP | Phenogroup 2 | Median: 0.42 | CV death | RV-PA uncoupling; PG2 has highest CV mortality (6.2%) |  |
| Restrictive LVDD (Grade 2–3) | Phenogroups 2 & 3 | ~55–65% | CV death | Advanced diastolic dysfunction associated with poor prognosis in PG2–3 |  |

*Abbreviations: AF, atrial fibrillation; CAD, Coronary artery disease; CKD, chronic kidney disease; COPD, Chronic obstructive pulmonary disease; CV, Cardiovascular; CVD, cerebrovascular disease; LVDD, Left Ventricular end-Diastolic diameter; PAD, peripheral artery disease; PASP, Pulmonary Artery Systolic Pressure; PG, Phenogroup; TAPSE, Tricuspid Annular Plane Systolic Excursion.*
